# Physical Activity and Its Association with Gestational Diabetes Mellitus Among Pregnant Women in Saudi Arabia: A Cross-Sectional Study

**DOI:** 10.3390/jcm15135263

**Published:** 2026-07-06

**Authors:** Samiha M. I. Abdelkader, Rehab F. M. Gwada, Saad A. Alhammad, Abdulfattah S. Alqahtani, Maha F. Algabbani, Fatimah A. Alsayegh

**Affiliations:** 1 Department of Rehabilitation Health Sciences, College of Applied Medical Sciences, King Saud University, Riyadh P.O. Box 10219, Saudi Arabia; sabdelkader@ksu.edu.sa (S.M.I.A.);; 2 Physical Therapy Department, National Heart Institute, Giza 12613, Egypt; 3 Department of Medical Rehabilitation, First Health Cluster, Riyadh 11622, Saudi Arabia

**Keywords:** physical activity, physical exercise, gestational diabetes mellitus, pregnancy, Saudi Arabia

## Abstract

**Background:** Gestational diabetes mellitus (GDM) is one of the most common medical complications of pregnancy and has a high prevalence in Saudi Arabia. GDM increases the risk of adverse maternal and fetal outcomes and is associated with cardiovascular risk factors. Glycemic severity, represented by the continuous 1 h plasma glucose value from the diagnostic two-hour 75 g oral glucose tolerance test (OGTT), may provide additional insight into glycemic status among women with GDM. Physical activity (PA) plays a vital role in maternal health. Therefore, the aim of this study was to identify the associations between PA and glycemic severity among pregnant women with GDM in Saudi Arabia and to identify factors associated with glycemic severity. **Methods**: This cross-sectional study enrolled 96 pregnant women during routine second-trimester visits at a maternity clinic in Riyadh. PA was assessed using the Pregnancy Physical Activity Questionnaire (PPAQ). Glycemic severity was assessed using the continuous 1 h plasma glucose value obtained from the diagnostic two-hour 75 g OGTT. **Results:** One-way ANOVA demonstrated a significant association between PA levels and glycemic severity (F = 2.78; *p* < 0.04). Multiple linear regression identified low-intensity PA, non-employment, and smoking during pregnancy were significantly associated with higher glycemic severity (*p* < 0.05). **Conclusions:** The study identifies a significant association between PA and glycemic severity. Furthermore, employment status and smoking were also significantly associated with glycemic severity. These findings suggest that PA and other modifiable lifestyle factors play role in glucose regulation during pregnancy. However, the cross-sectional design precludes any inference of causality.

## 1. Introduction

Gestational diabetes mellitus (GDM) is one of the most common medical complications of pregnancy. It is a type of diabetes mellitus characterized by maternal hyperglycemia and defined as any stage of glucose intolerance that develops during pregnancy or is first recognized during pregnancy [1]. The prevalence of GDM is rising globally [2]. With particularly high rates of GDM reported in Saudi Arabia (SA), where it affects up to 32.6% of pregnant women in the Riyadh region [3].

GDM substantially increases the risks of complications for both mother and fetus, which may lead to excess fetal growth and impaired glucose metabolism [3,4]. Furthermore, it is associated with maternal cardiovascular risk factors such as hypertension, dyslipidemia, and obesity [5]. Women with a history of GDM have a significantly higher predicted postpartum risk of major cardiovascular events within the first to third decade after delivery [6].

The degree of maternal hyperglycemia among women with GDM may have important clinical implications, as higher maternal glucose concentrations have been associated with an increased risk of adverse maternal outcomes. Furthermore, evidence suggests a graded relationship between oral glucose tolerance test glucose values and pregnancy outcomes, highlighting the importance of evaluating glycemic severity as an indicator of glycemic burden among women diagnosed with GDM [7].

Physical activity (PA), defined as any energy-expending bodily movement produced by skeletal muscles, is essential for promoting maternal and fetal health and enhances both physiological and physical outcomes. An increase in PA levels among pregnant women would enhance their cardiorespiratory health, reduce anxiety and depression, reduce excessive weight, improve body image satisfaction, lower the risk of GDM and hypertensive disorders, and improve quality of life in healthy pregnant women [8,9,10].

Regarding fetal health, PA enhances maternal cardiovascular function and tissue oxygenation, creating a more favorable intrauterine environment, potentially through preserved placental vascular function and a reduced risk of fetal overgrowth (macrosomia) [8,9,10].

Evidence suggests that PA plays an important role in maternal glucose regulation during pregnancy. Prospective study has shown that higher levels of habitual PA are associated with improved glycemic control across pregnancy [11]. Similarly, among women with GDM, greater engagement in PA has been linked to improved achievement of glycemic targets [12]. Moreover, recent systematic reviews have confirmed that exercise interventions during pregnancy improve glycemic outcomes and reduce adverse metabolic consequences [13].

Despite the critical role of PA, a large proportion of Saudi women reduce their PA during pregnancy, with many transitioning from an active to a sedentary lifestyle [10]. This decrease is attributed to a complex interplay of cultural, social, and environmental factors. Identified barriers include lack of awareness regarding the importance of exercise, fatigue, childcare responsibilities, lack of motivation, limited access to appropriate exercise facilities, and the constraint of extreme weather conditions [10,14].

Given the exceptionally high prevalence of GDM in SA (32.6%) and the need for early prevention and management [15], understanding modifiable risk factors is essential. However, there is a significant gap in the national research concerning the specific association between PA and glycemic severity among Saudi pregnant women. To develop effective, culturally appropriate interventions that may decrease glycemic severity, this association must be clearly established within the local context.

Therefore, the aim of this study was (1) to identify the associations between PA and glycemic severity among pregnant women with GDM in SA, operationalized as the continuous 1 h plasma glucose value obtained from the diagnostic two-hour 75 g oral glucose tolerance test (OGTT), and (2) to identify factors associated with glycemic severity.

Based on previous evidence suggesting associations between PA and glycemic severity during pregnancy, we hypothesized that higher levels of PA would be associated with lower glycemic severity among women with GDM. We further hypothesized that selected factors, including age, educational level, working status, parity, pre-pregnancy body mass index (BMI), gestational weight gain (GWG), and smoking during pregnancy, would be associated with glycemic severity.

## 2. Methods

### 2.1. Study Design and Setting

An analytical cross-sectional design was used for this study. A self-administered online questionnaire was used to collect the data. Pregnant women were voluntarily enrolled from the maternity clinics in the Obstetrics and Gynecology Department at King Khalid University Hospital in Riyadh, SA. Data collection took place over five months, within online questionnaires distributed and returned between June 2023 and November 2023.

### 2.2. Participants

Pregnant women diagnosed with GDM at 24 to 28 weeks’ gestation (second trimester), with singleton pregnancy (single fetus), and aged between 18 and 40 years were included. Participants were excluded if they had preexisting chronic systemic diseases, such as cardiorespiratory diseases, diabetes mellitus, hypertension requiring medication, and chronic renal disease; if they had physical limitations that prevented them from doing PA; if they had mental limitations that prevented them from understanding questioners or communication; and if they had contraindications to exercise, such as heart or lung diseases, cervical cerclage, twin or higher pregnancy, placenta previa after 26 weeks of pregnancy, preterm labor, or ruptured membranes during pregnancy.

### 2.3. Sample Size

The required sample size was calculated using the Correlation Sample Size Calculator (https://sample-size.net/correlation-sample-size/, accessed on 9 June 2026) to determine whether the correlation coefficient between PA and glycemic severity differed significantly from zero. A two-tailed significance level of 0.05 (α = 0.05) and a statistical power of 80% (β = 0.20) were applied. The expected correlation coefficient was set at r = 0.30, based on evidence from previous systematic reviews and meta-analyses demonstrating a moderate association between PA and GDM risk [16]. The minimum required sample size was estimated to be 85 participants.

### 2.4. Ethical Approval and Participant Consent

The ethical approval for this study (no. E-23-7586, in 15 March 2023) was obtained from the Institutional Review Board of the College of Medicine, King Saud University. Participants signed a written informed consent form after receiving information about the study’s aims and procedures.

### 2.5. Study Procedures

Pregnant women who met the eligibility criteria were identified during routine second-trimester check-ups. Once diagnosed with GDM, eligible participants were referred by physicians in the Department of Obstetrics and Gynecology to the researcher. The study’s objectives and procedures were explained to the participants, including the purpose and process of the research. Subsequently, the questionnaire was provided to them for completion. Clinical data related to glycemic severity, including two-hour 75 g oral glucose tolerance test values, were extracted from the participants’ medical records.

### 2.6. Instruments

Screening for GDM was performed as part of routine antenatal care using the universal two-hour 75 g oral glucose tolerance test (OGTT). GDM was diagnosed according to World Health Organization (WHO) criteria, defined by a 1 h plasma glucose level ≥ 10.0 mmol/L [17]. The laboratory results were obtained retrospectively from participants’ medical records; no laboratory tests were conducted by the researcher. In the present study, glycemic severity among women with GDM was operationalized using the continuous 1 h plasma glucose concentration value obtained during the diagnostic two-hour 75 g OGTT. This value was used as a surrogate measure of glycemic severity for the statistical analyses.

A self-administered questionnaire consisting of two sections was used to collect the study data. The first section included socio-demographic and pregnancy-related information, including age (years old), level of education (elementary, intermediate, high school, or university studies), working status (employee or not), and pregnancy health (gestational week, parity, smoking during pregnancy, singleton pregnancy or multiple gestations, and comorbidities). Participants also self-reported their pre-pregnancy weight and height, which were used to calculate pre-pregnancy body mass index (pre-pregnancy BMI). Current pregnancy weight was also self-reported and used to calculate gestational weight gain (GWG). The second section consisted of the Pregnancy Physical Activity Questionnaire (PPAQ).

### 2.7. Pregnancy Physical Activity Questionnaire (PPAQ)

PPAQ is a self-reported questionnaire to assess PA practices during pregnancy in the preceding three months. It is valid and reliable in Arabic [18]. It provides a comprehensive assessment of PA, having 31 questions, including 14 activities dealing with household and caregiving, 3 concerning transportation, 9 sports-related activities, and 5 occupational activities. There are two open-ended questions concerning sport-type activities, allowing the responder to specify types not listed previously and practiced on a weekly basis [19].

PA levels were calculated according to the scoring approach described in the validated Arabic version of the PPAQ. Each self-reported activity was assigned its corresponding metabolic equivalent (MET) value and multiplied by the reported duration to obtain energy expenditure expressed as MET-hours/day. The resulting values were summed across all questionnaire items and converted into a mean daily energy expenditure score. Participants were subsequently categorized into sedentary (<1.5 METs), light-intensity (1.5–<3.0 METs), moderate-intensity (3.0–6.0 METs), and vigorous-intensity (>6.0 METs) PA groups, as previously described in the Arabic PPAQ validation study [18].

### 2.8. Statistical Analysis

Data obtained from laboratory tests and questionnaires were reviewed, coded, and entered into Microsoft Excel 2016 (Microsoft Corp., Redmond, WA, USA). The completed dataset was subsequently imported into SPSS version 26.0 (IBM Corp., Armonk, NY, USA) for statistical analysis.

Descriptive statistics were presented as mean and standard deviation (SD) for normally distributed continuous variables and as median and interquartile range (IQR) for non-normally distributed continuous variables. Categorical data were presented as frequencies and percentages.

An association between PA levels and glycemic severity, measured as the continuous 1 h plasma glucose value obtained from the diagnostic two-hour 75 g OGTT, was assessed by comparing mean glucose values across PA levels using one-way analysis of variance (ANOVA).

Referring to previous studies that identified maternal demographic and lifestyle characteristics as important factors associated with glycemic severity during pregnancy [20], multiple linear regression analysis was performed using the continuous 1 h OGTT plasma glucose value as the dependent variable to identify independent associated factors of glycemic severity. The independent variables included age, PA, educational level, parity, working status, pre-BMI, GWG, and smoking during pregnancy. Continuous variables (age and GWG) were entered into the regression model as continuous measures. Binary categorical variables (working status, parity, and smoking during pregnancy) were entered binary variables, whereas ordinal variables (physical activity, educational level, and pre-pregnancy BMI) were entered according to their natural ordering. Regression coefficients (β), 95% confidence intervals (CIs), and *p*-values were reported. *p*-values < 0.05 were considered statistically significant.

Prior to regression analysis, the assumptions of multiple linear regression were assessed. Normality of residuals was evaluated using histograms and normal P–P plots. Linearity and homoscedasticity were assessed by visual inspection of scatterplots of standardized residuals versus standardized predicted values. Independence of errors was evaluated using the Durbin–Watson statistic (2.10). Multicollinearity was evaluated using tolerance values (>0.20) and variance inflation factors (VIF < 5). Model fit was evaluated using the coefficient of determination (R^2^), adjusted R^2^, and the overall F-test. Influential observations were assessed using standardized residuals and Cook’s distance, and no serious violations of the regression assumptions were identified.

## 3. Results

The study included 96 pregnant women diagnosed with GDM. The median age of study participants was 33 years (IQR: 8). Most participants held a bachelor’s degree (64%) and were not employees (76%). A substantial majority of the women were multiparous (72.9%).

Regarding clinical metrics, the mean concentration from the two-hour 75 g oral glucose tolerance test was 10.69 (±0.89) mmol/L. A total of 40.6% of the cohort was classified as having a sedentary PA level. Furthermore, 41.7% had pre-pregnancy obesity and a mean GWG of 5 kg during pregnancy. They are shown in Table 1.

Table 2 presents the mean 1 h OGTT glucose values across PA levels. Participants classified as sedentary exhibited the highest mean glucose value (10.93 ± 0.76 mmol/L), followed by those with the low-intensity (10.74 ± 1.00 mmol/L), moderate-intensity (10.33 ± 0.89 mmol/L), and vigorous-intensity (10.20 ± 0.62 mmol/L) PA levels. A decreasing trend in mean glucose values was observed with increasing PA levels.

### 3.1. Association Between Physical Activity Levels and Glycemic Severity

**Figure 1:**
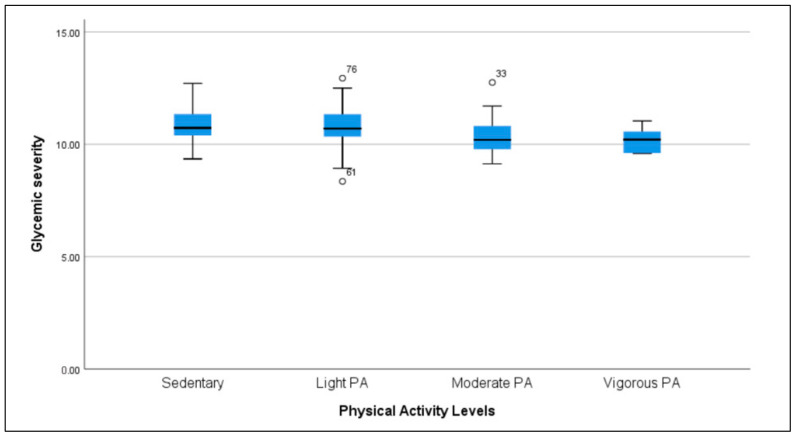
Box plots of glycemic severity by physical activity levels. The central line represents the median, boxes show the interquartile range (IQR), whiskers indicate the minimum and maximum values, and dots represent outliers. One-way ANOVA indicated a significant association, with mean glycemic severity across physical activity level.

A one-way ANOVA was conducted to assess the association between PA levels and glycemic severity. Homogeneity of variances was confirmed (Levene’s test, *p* = 0.704). Results indicated a significant association, with mean glycemic severity differing across the PA levels (F = 2.78, *p* = 0.04) (as shown in Table 3 and Figure 1).

The effect size was moderate (η^2^ = 0.083), indicating that approximately 8.3% of the variance in glycemic severity was explained by differences in PA levels.

Post hoc pairwise comparisons were conducted using Tukey’s HSD and Bonferroni tests following the significant overall ANOVA result. Although a significant overall difference in glycemic severity was observed across PA levels, no statistically significant pairwise differences were identified after adjustment for multiple comparisons (all *p* > 0.05). The comparison between sedentary and moderate-intensity PA levels showed a trend toward statistical significance (Tukey *p* = 0.054; Bonferroni *p* = 0.06).

### 3.2. Analysis of Factors Associated with Glycemic Severity Among Women with Gestational Diabetes Mellitus

Factors associated with glycemic severity, including age, PA, educational level, working status, parity, pre-BMI, GWG, and smoking during the second trimester of pregnancy, were examined using multiple linear regression analysis (Table 4).

The multiple linear regression model was statistically significant (F(8,87) = 2.738, *p* = 0.01). The model explained 20.1% of the variance in glycemic severity (R^2^ = 0.20), with an adjusted R^2^ of 0.12. The Durbin–Watson statistic was 2.10, indicating no substantial autocorrelation of residuals.

Pregnant women who engaged in low-intensity PA predicted a significantly higher glycemic severity (β: −0.37; 95% CI: −0.60–−0.14; *p* < 0.05). Additionally, non-employed participants and those who reported smoking during pregnancy demonstrated significantly increased probabilities of glycemic severity (β: 0.56 and β: 0.61, and 95% CI: 0.10–1.03 and 95% CI: 0.11–1.12, respectively; *p* < 0.05).

No significant associations were observed between glycemic severity and the rest of the factors: maternal age, educational level, parity, pre-BMI, and GWG (β: 0.02, β: −0.008, β: −0.26, β: −0.26, β: 0.61; 95% CI: −0.01–0.06, 95% CI: −0.26–0.25, 95% CI: −0.65–0.13, 95% CI: −0.26–0.26, 95% CI: −0.09–0.08; *p* > 0.05).

## 4. Discussion

This study aimed to examine the association between PA and glycemic severity among pregnant women with GDM in SA and to identify factors associated with glycemic severity. The key finding was a significant association between PA levels and glycemic severity.

In addition, after adjusting for factors including age, PA, educational level, parity, working status, pre-BMI, GWG, and smoking during pregnancy, significant associations were observed between PA, working status, and smoking with glycemic severity. Specifically, non-employed women, those engaging in low-intensity PA, and those who reported smoking during pregnancy were associated with higher glycemic severity.

The present findings are consistent with a previous meta-analysis that reported a significant association between PA during the second trimester and glycemic outcomes. Although most previous studies focused on the risk of developing GDM rather than glycemic severity among women already diagnosed with GDM, higher levels of PA have consistently been associated with more favorable glucose regulation and metabolic health during pregnancy [10,21]. Therefore, the current findings suggest that PA may also be associated with variation in glycemic severity among women with GDM.

Several biological mechanisms may partly explain this association. Previous research has suggested that PA may improve insulin sensitivity and glucose utilization while reducing oxidative stress and inflammatory processes involved in the pathophysiology of GDM [10,21]. In addition, PA may support β-cell function and contribute to improved glucose regulation. Although these mechanisms were not directly assessed in the present study, they provide biologically plausible explanations for the observed association between higher PA levels and lower glycemic severity [10,21].

The present findings also suggest that glycemic severity may be influenced by additional maternal characteristics. Previous studies have reported associations between glycemic severity and factors such as obesity, advanced maternal age, family history of diabetes, and lifestyle-related factors [22,23,24,25]. While the specific factors identified in the current study differed from those reported previously, the findings support the multifactorial nature of glycemic regulation during pregnancy and highlight the potential contribution of several factors to glycemic severity among women with GDM.

In contrast, some previous studies reported no significant association between PA and glycemic severity during pregnancy. Differences in study populations, timing of PA assessment, PA measurement methods, and intervention characteristics may partly explain these inconsistent findings. In addition, some studies have suggested that the effectiveness of PA may depend on pre-pregnancy lifestyle behaviors and underlying metabolic status. Furthermore, dietary factors, which were not assessed in the present study, may influence glycemic outcomes and potentially modify the relationship between PA and glycemic severity [26,27,28]. Further longitudinal and intervention studies are needed to better understand the role of PA in glycemic severity among women with GDM.

Overweight and obesity are recognized as important public health concerns that may adversely affect maternal health during pregnancy [22]. Previous studies have suggested that PA during pregnancy may help reduce GWG and improve glycemic outcomes through enhanced glucose uptake by skeletal muscle and improved insulin sensitivity [21,24,29]. Furthermore, some studies have reported that higher pre-pregnancy BMI may modify the association between physical activity and glycemic severity, with overweight or obese women potentially deriving different benefits from exercise interventions compared with normal-weight women [30].

However, in the present study, neither pre-pregnancy BMI nor gestational weight gain was significantly associated with glycemic severity. This discrepancy may be related to differences in study populations, sample size, assessment methods, or the clinical characteristics of the participants. As all women in the current study had GDM, the limited variability in glycemic status may have attenuated the associations observed in previous studies.

Another important finding of the present study was that non-employed women exhibited higher glycemic severity than employed women. Although evidence directly examining the association between employment status and glycemic severity among women with GDM remains limited, previous studies have suggested that occupational and socioeconomic factors may influence gestational glycemic outcomes. Previous research has reported that non-employed pregnant women had a significantly higher risk of developing GDM compared with employed women, highlighting the potential role of employment status in maternal metabolic health [31,32].

Furthermore, employed women with GDM have been reported to demonstrate greater healthy-living awareness than unemployed women. These findings suggest that occupational status may be related to health-related behaviors and self-management practices during pregnancy, which may partly explain the higher glycemic severity observed among non-employed women in the present study [33]. However, the mechanisms underlying this association were not assessed and require further investigation.

Smoking was associated with glycemic severity, consistent with Lü et al.’s (2021) findings among pregnant women [34]. Although the present study did not examine the effect of smoking on glycemic severity, these findings suggest that comorbid substance use may influence maternal health outcomes during pregnancy.

Smoking was associated with glycemic severity, consistent with the findings of Lü et al. (2021) among pregnant women [34]. Previous studies have suggested that smoking may adversely affect maternal glucose metabolism and contribute to unfavorable glycemic outcomes during pregnancy. In addition, smoking has been linked to increased oxidative stress and inflammatory responses, both of which have been implicated in the pathophysiology of GDM and insulin resistance [35,36]. These biological processes may impair glucose regulation and contribute to poorer glycemic control. Although these mechanisms were not directly assessed in the present study, they provide biologically plausible explanations for the observed association between smoking and higher glycemic severity. Furthermore, evidence from recent systematic reviews indicates that exposure to tobacco smoke, including passive smoking, is associated with an increased risk of GDM, further supporting the potential impact of smoking-related factors on maternal glycemic health [37].

## 5. Strengths and Limitations

This study focused on pregnant women diagnosed with GDM in SA, targeting a specific demographic that may be underrepresented in the international literature. This approach enhances contextual relevance and addresses a knowledge deficiency regarding PA and glycemic severity in this population. Additionally, recruiting participants at the time of GDM diagnosis allowed for the assessment of PA and associated factors in proximity to the clinical event, thereby minimizing recall bias and strengthening the reliability of self-reported data.

This study has some limitations. Its cross-sectional design and recruitment from a single hospital in Riyadh restrict causal inference and generalizability. Additionally, the reliance on self-reported measures, an age limit of 40 years, and a relatively small sample size may have influenced the findings, although the instruments were validated and the sample was sufficient to test the associations.

## 6. Clinical Implications

The observed associations between PA and glycemic severity among pregnant women with GDM indicate the need for culturally appropriate education programs in SA that address misconceptions, barriers related to PA and exercise during pregnancy, and exercise recommendations that may enhance maternal engagement and adherence. These programs could be designed to empower women with the knowledge and confidence to engage in safe levels of PA. Incorporating PA guidance requires collaboration between obstetricians, endocrinologists, physiotherapists, and health educators.

Although GDM typically resolves after delivery, affected women remain at increased risk of recurrence in subsequent pregnancies and of developing type 2 diabetes mellitus [38,39]. The associations observed in this study support the relevance of incorporating PA counseling into routine prenatal care for women with GDM. These findings may help inform the development of tailored exercise programs and lifestyle interventions for this population. However, longitudinal and interventional studies are needed to determine whether increasing PA leads to improvements in glycemic outcomes and long-term metabolic health.

## 7. Conclusions

The study concluded that there was a significant association between PA and glycemic severity among pregnant women. In addition, PA, working status, and smoking were identified as factors significantly associated with glycemic severity. These findings suggest that factors provide insight linked to glycemic severity and highlight the potential relevance of maintaining adequate levels of PA in the context of glycemic severity and maternal metabolic health. However, due to the cross-sectional design, no causal relationships can be inferred.

## 8. Recommendations and Future Directions

It is recommended to conduct comparative studies between pregnant women with and without GDM to assess their PA. Additional research with similar objectives should be performed, incorporating various regions of SA. Furthermore, the association between PA and GDM should be examined using objective measures of PA, such as accelerometers.

## Figures and Tables

**Table 1:** Characteristics of pregnant participants.

Study Group (*n* = 96)		
Variables		^a^ Values
Age (years)		33 (IQR 8)
Academic level	Elementary	1 (1%)
	Intermediate	3 (3.1%)
	High school	21 (21.9%)
	Bachelors	62 (64.6%)
	Masters’	7 (7.3%)
	Ph.D.	2 (2.1%)
Working status	Employee	23 (24%)
	Not employee	73(76%)
Gestational week	24	6 (6.3%)
	25	9 (9.4%)
	26	16 (16.7%)
	27	25 (26%)
	28	40 (41.7%)
Parity	Nulliparous	26 (27.1%)
	Multiparous	70 (72.9%)
Smoking habit	Smoked	14 (14.6%)
	Not smoked	82 (85.4%)
Pre-BMI (Kg/m^2^)	Underweight	1 (1%)
	Normal weight	20 (20.8%)
	Overweight	35 (36.5%)
	Obese	40 (41.7%)
GWG (Kg)		5 (±0.20)
OGTT (mmol/L)	1 h plasma glucose value	10.69 (±0.89)
PPAQ level (METs)	Sedentary activity	39 (40.6%)
	Low-intensity PA	29 (30.2%)
	Moderate-intensity PA	23 (24.0%)
	Vigorous-intensity PA	5 (5.2%)

*n*: number of participants. OGTT: Two-hour 75 g oral glucose tolerance test; Pre-BMI: pre-pregnancy body mass index; GWG: gestational weight gain; PPAQ: Pregnancy Physical Activity Questionnaire; PA: physical activity. ^a^ Values are mean (±standard deviation) for normally distributed continuous variables, median (interquartile range) for non-normally distributed continuous variables, or frequencies (percentage, %) for categorical variables.

**Table 2:** One-hour OGTT glucose values according to physical activity levels.

Physical Activity Levels	*n*	Mean	Standard Deviation
Sedentary	39	10.93	0.76
Low-intensity	29	10.74	1.00
Moderate-intensity	23	10.33	0.89
Vigorous-intensity	5	10.20	0.62

*n*: number of participants.

**Table 3:** Association between physical activity levels and glycemic severity according to a one-way ANOVA.

Variable	One-Way ANOVA		
Glycemic Severity (mmol/L)	Mean Square	F	*p*-Value
	2.12	2.78	0.04 *

* Significant at *p* < 0.05. Effect size (η^2^) = 0.083, indicating a moderate effect.

**Table 4:** Multiple linear regression analysis of factors associated with glycemic severity.

Variables	*p*-Value	β (95% CI)
Physical activity	0.002 *	−0.37 (−0.60–−0.14)
Age	0.30	0.02 (−0.01–0.06)
Education	0.95	−0.008 (−0.26–0.25)
Working status	0.01 *	0.56 (0.10–1.03)
Parity	0.19	−0.26 (−0.65–0.13)
Pre-pregnancy BMI	0.98	−0.002 (−0.26–0.26)
GWG	0.89	−0.006 (−0.09–0.08)
Smoking	0.01 *	0.61 (0.11–1.12)

Abbreviation: β = unstandardized regression coefficient (B); CI: confidence interval; Sig: statistically significant; Pre-BMI: pre-pregnancy body mass index; GWG: gestational weight gain.* Significant at *p* < 0.05. The overall regression model was statistically significant (F(8,87) = 2.73, *p* = 0.01), explaining 20.1% of the variance in glycemic severity (coefficient of determination (R^2^) = 0.20; adjusted R^2^ = 0.12).

## Data Availability

The data presented in this study are available from the corresponding author upon reasonable request. The data are not publicly available due to their containing information that could compromise the privacy of the research participants.
